# Assessing the Role of EEG Biosignal Preprocessing to Enhance Multiscale Fuzzy Entropy in Alzheimer’s Disease Detection

**DOI:** 10.3390/bios15060374

**Published:** 2025-06-10

**Authors:** Pasquale Arpaia, Maria Cacciapuoti, Andrea Cataldo, Sabatina Criscuolo, Egidio De Benedetto, Antonio Masciullo, Marisa Pesola, Raissa Schiavoni

**Affiliations:** 1 Department of Information Technology and Electrical Engineering, University of Naples Federico II, 80125 Naples, Italymaria.cacciapuoti@unina.it (M.C.); sabatina.criscuolo@unina.it (S.C.); marisa.pesola@unina.it (M.P.); 2 Institute of Intelligent Industrial Technologies and Systems for Advanced Manufacturing, National Research Council of Italy, 20133 Milan, Italy; 3 Department of Engineering for Innovation, University of Salento, 73100 Lecce, Italy; andrea.cataldo@unisalento.it (A.C.); antonio.masciullo@unisalento.it (A.M.); raissa.schiavoni@unisalento.it (R.S.)

**Keywords:** Alzheimer’s disease, electroencephalography, signal processing, multiscale fuzzy entropy, complexity, bioimaging, health monitoring

## Abstract

Quantitative electroencephalography (QEEG) has emerged as a promising tool for detecting Alzheimer’s disease (AD). Among QEEG measures, Multiscale Fuzzy Entropy (MFE) shows great potential in identifying AD-related changes in EEG complexity. However, MFE is intrinsically linked to signal amplitude, which can vary substantially among EEG systems, and this hinders the adoption of this metric for AD detection. To overcome this issue, this study investigates different preprocessing strategies to make the calculation of MFE less dependent on the specific amplitude characteristics of the EEG signals at hand. This contributes to generalizing and making more robust the adoption of MFE for AD detection. To demonstrate the robustness of the proposed preprocessing methods, binary classification tasks with Support Vector Machines (SVMs), Random Forest (RF), and K-Nearest Neighbor (KNN) classifiers are used. Performance metrics, such as classification accuracy and Matthews Correlation Coefficient (MCC), are employed to assess the results. The methodology is validated on two public EEG datasets. Results show that amplitude transformation, particularly normalization, significantly enhances AD detection, achieving mean classification accuracy values exceeding 80% with an uncertainty of 10% across all classifiers. These results highlight the importance of preprocessing in improving the accuracy and the reliability of EEG-based AD diagnostic tools, offering potential advancements in patient management and treatment planning.

## 1. Introduction

Alzheimer’s disease (AD) is a neurodegenerative condition primarily affecting individuals over the age of 65 [1]. Nowadays, the accurate and objective diagnosis of AD is a significant challenge in the field of neurology. Neuropsychological tests, such as the mini-mental state examination [2], can be useful for assessing the severity of cognitive impairment, and for identifying the specific cognitive area affected [1,3]. However, these assessments usually provide a preliminary diagnosis that must be corroborated with biomarkers obtained through neuroimaging techniques like magnetic resonance imaging (MRI) and positron emission tomography (PET), as well as cerebrospinal fluid analysis [1,4]. Despite their clinical acceptance, these techniques have several drawbacks, including high costs, low temporal resolution, exposure to radiation, and invasiveness [5].

As a result, currently, there has been a growing interest in utilizing electroencephalography (EEG) signals for detecting, monitoring, and predicting AD [4,6,7]. EEG represents a low-cost, wearable, non-invasive, easy-to-access method of analyzing the brain’s electrical activities with a high temporal resolution [8,9]. In particular, quantitative EEG (QEEG) employs numerical and statistical measures to provide objective representations of brain activity [10].

In both clinical and research applications, linear metrics such as time–frequency analysis and event-related potentials are frequently employed [4,6]. However, the brain is a non-stationary and complex structure; hence, linear metrics alone are insufficient for capturing its full dynamics [11,12]. The complexity analysis of EEG signals has the potential to supplement traditional analysis techniques, and it offers greater sensitivity in identifying complex neuronal processes within the brain and biomarkers of psychopathology such as depression, schizophrenia, and neurodegenerative disorders [12,13,14]. Among the various methods used to assess complexity, entropy has emerged as a key metric. Entropy, in the context of EEG signals, refers to the degree of unpredictability or disorder within the signal [12]. High entropy typically indicates a more complex, less predictable signal, while low entropy suggests a more regular or predictable signal. With regard to AD, several studies in the literature have shown that this disease is associated with a loss of EEG entropy [12,15,16]. Among the entropy metrics, Multiscale Fuzzy Entropy (MFE) has emerged as an indicator of AD [17,18]. However, despite its potential, there is currently no recognized method for applying MFE to EEG signals: this has limited the possibility of comparison between studies’ results [19,20] and, ultimately, has prevented MFE from becoming a reliable indicator of AD. Typically, EEG signals are processed for the removal of artifacts, filtering in the bands of interest, and division into epochs [21]. Nevertheless, the MFE formula is intrinsically linked to signal amplitude [22]. This is particularly relevant when working with EEG data. As a matter of fact, one major obstacle in EEG analysis is the variability in EEG signal acquisition, both within and between subjects. This variability can be attributed to factors such as electrode impedance, amplifier gain, and the configuration of acquisition systems, which can substantially affect the amplitude of EEG signals and introduce bias. Consequently, the extraction of meaningful features from the EEG data is hindered. In the context of MFE, this variability can potentially lead to biased distances calculated on embedded vectors towards data with larger amplitude ranges [22,23].

Building on these considerations, this study introduces the application of amplitude transformation techniques as a critical preprocessing step for EEG signal analysis. These transformations are specifically designed to mitigate both intra- and inter-subject variability arising from differences in EEG acquisition systems, thereby minimizing the influence of system-specific characteristics on the MFE calculation. The primary objective is to underscore the pivotal role of amplitude transformation in enhancing the reliability of MFE-based biomarkers for AD detection. By standardizing the amplitude characteristics of the EEG signals, this approach facilitates more consistent and reliable comparisons across subjects, ensuring that the extracted features predominantly reflect the underlying neurophysiological activity of the brain, rather than being confounded by technical discrepancies in signal acquisition.

To evaluate the impact of EEG amplitude transformation in MFE-based AD detection, EEG signals from patients with AD and healthy subjects (HSs) are considered. MFE is extracted from the EEG signals as a feature and is used in a binary classification task to distinguish between HS and AD. Different machine learning (ML) approaches are applied, including Support Vector Machines (SVMs), Random Forest (RF), and K-Nearest Neighbor (KNN). The performance of the models is evaluated by considering the classification accuracy and Matthew Correlation Coefficient (MCC). To validate the method and demonstrate its generalizability, the EEG data from two publicity datasets are employed. In this way, the robustness of the proposed approach is assessed by taking into account various EEG acquisition systems and protocols. This ensures that the effects observed are not dependent on the specific equipment or dataset but reflect genuine differences in neurophysiological activity related to AD. Our results demonstrate that amplitude transformation—especially through normalization—significantly enhances the detection of AD, reducing both intra- and inter-subject variability. This preprocessing step helps to create a level playing field for EEG features, enabling more precise and reliable comparisons across subjects.

This study not only addresses the need for a robust and effective preprocessing pipeline in EEG analysis [10] but it also demonstrates the practical impact of amplitude transformation on the effectiveness of MFE as a biomarker for AD. By establishing a reproducible and generalized preprocessing procedure, this work paves the way for future studies to achieve more robust and comparable results, thereby enhancing the diagnostic utility of EEG-based methods in neurodegenerative disease research.

The paper is structured as follows. Section 2 presents an overview of MFE complexity analysis in AD. Section 3 outlines the proposed method, describing the adopted EEG amplitude transformations. Section 4 presents the experimental approach for the method validation. The obtained results and the discussion are reported in Section 5. Finally, conclusions and future work are outlined in Section 6.

## 2. Background and Motivation

Complexity measures quantify the irregularity and variability of the informational content within a signal, encompassing both temporal and spatial dimensions. Two essential attributes of a complex system are *predictability* and *regularity*, which together provide an understanding of its dynamic behavior [12].

Measures of regularity, such as entropy metrics, are frequently employed to detect recurring patterns in time series data [12,17,24]. The application of the concept of entropy to EEG represents a statistical methodology for quantifying the degree of randomness in a time series. Entropy metrics are commonly employed in the field of anesthesia, demonstrating a reduction in complexity with the administration of anesthesia [25], which may be indicative of the increased predictability or repetitiveness of the EEG signal. Furthermore, entropy has been employed for the analysis of pathological states, including schizophrenia, depression, and neurodegenerative disorders such as AD [12]. Indeed, several complexity-based EEG biomarkers have been employed to examine brain dynamics in AD, revealing significant alterations compared to control groups [4,17,26,27].

In recent years, there has been a significant increase in the use of multiscale entropy formulations in EEG analysis. In fact, the analysis of EEG signals at different spatial and temporal scales can provide valuable insights into brain dynamics. Accordingly, a multiscale approach enables a comprehensive understanding of the complexity of the EEG brain signal in both the spatial and temporal domains.

With regard to AD, multiscale entropy formulations have demonstrated a high sensitivity to the severity of the disease, indicating a decline in complexity as the disease progresses from moderate to severe stages [22,27,28,29]. Among the multiscale approaches, MFE, based on Fuzzy Entropy, has emerged as a powerful tool in AD detection [17].

Fuzzy Entropy quantifies the regularity or chaos of a time series by analyzing the signal through a sliding window that searches for similar patterns. More specifically, it uses a sliding window technique to examine signals, identifying similar patterns and estimating the probability that a subseries of length (*m*) will maintain its similarity when extended to (m+1) [17]. Fuzzy Entropy is based on the theory of fuzzy logic, which allows for a continuous degree of truth, with values ranging from 0 (representing “totally false”) to 1 (representing “totally true”). These continuous values can be interpreted as a degree of belonging to a set of similar patterns [17].

The MFE represents an extension of the Fuzzy Entropy concept. The rationale behind the multiscale approach is to recalculate the entropy on the original signal each time the time scale is varied [17]. Fuzzy Entropy and its multiscale formulation are used to analyze EEGs from AD patients and HS, revealing lower fuzzy entropy values in AD patients and demonstrating superior diagnostic accuracy compared to other entropy measures [30]. In [18], MFE is combined with connectivity metric to classify EEG data from AD and HS, with accuracy higher than 80%. In [17], MFE allows discrimination between AD and HS across multiple time scales, finding that patients with AD exhibit elevated complexity values for the slow frequency bands in comparison to HS, whereas the opposite is observed for the fast frequency bands.

As mentioned in Section 1, although MFE complexity analysis anticipates good potential in investigating neurophysiological mechanisms, there is no standardized approach for applying MFE to EEG signals, making it difficult to compare studies and to generalize the adoption of MFE as an indicator for AD [20,21]. Typically, the preprocessing stages followed in EEG studies include artifact removal, signal filtering in the bands of interest, and signal division into epochs [21,31]. Nevertheless, the MFE formulation is inherently associated with signal amplitude, particularly in multivariate data such as EEG. Specifically, the fuzzy membership function is derived from the absolute distance between data points in a time series and its shifted version, following a coarse-graining process. This process involves averaging signal points within a window whose length is determined by the scale factor. Consequently, the amplitude of the signal directly influences this averaging process. This can result in the calculation of biased distances on embedded vectors towards data with larger amplitude ranges, which may lead to erroneous findings [22,23]. As a matter of fact, EEG signal amplitudes exhibit considerable variability, both between subjects and across different acquisition systems [10,32]. In this regard, amplitude transformation can mitigate intra- and inter-subject variability, improving the robustness of the analysis [20,32] and enabling meaningful comparisons of MFE across diverse EEG signals [33].

Although MFE has shown promising results in EEG-based analysis, the impact of preprocessing strategies—particularly amplitude transformation—on its estimation has not been systematically explored. In the literature, the term “normalization” is often used inconsistently or without clarification of the specific method adopted (e.g., min-max, z-score, per-channel, or global transformations). This absence of standardization impairs reproducibility and hampers direct comparison between studies.

In addition to this, there are studies showing how the classification performance of EEG signals changes with different preprocessing [20], and specifically, how classification performance varies with different EEG signal normalization, showing that classification performance varies depending on whether normalization is applied to the EEG signals [32]. However, existing studies have primarily focused on preprocessing EEG signals to remove artifacts or improve signal quality, but little attention has been given to how these transformations influence entropy-based biomarkers. Starting from these considerations, the present study addresses this gap by focusing on the preprocessing stage of EEG signals, systematically evaluating different clearly defined amplitude transformation techniques, based on min-max normalization and standardization.

This operation is crucial to mitigating issues arising from differences in data scales, thereby ensuring greater compatibility and improving both the effectiveness and interoperability of analytical models. In this context, this study opts for a univariate analysis approach, whereby the MFE is computed independently for each EEG channel. This choice allows to preserve channel-specific information and to explore how different amplitude transformations affect MFE values on a per-channel basis. While multivariate entropy methods can be valuable for capturing spatiotemporal patterns and network-level interactions across EEG channels [24,34,35], this work focuses on a more granular investigation. Specifically, the univariate approach allows preserving channel-specific information and to evaluate the overall impact of preprocessing without aggregating across channels.

Thus, considering EEG signals from AD and HS, different amplitude transformation approaches based on min-max normalization and standardization are investigated in order to assess their impact on MFE evaluation. The case in which no amplitude transformation is made is considered as the reference. By using the MFE extracted from EEG signals as features, a binary classification task is addressed to assess the impact of amplitude transformation in discriminating between HS and AD.

## 3. Method

**Figure 1:**
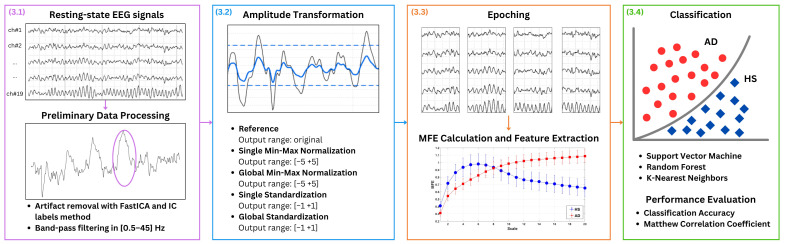
Proposed method. EEG signals from patients with AD and HS in closed-eye resting state condition are considered. The EEG signals are denoised by using Independent Component Analysis (ICA), utilizing the FastICA algorithm and IC labels with an empirical rejection threshold set at 90%. A representative example of an artifact removed through this procedure is highlighted by the purple circle in Figure 1. Then, the signal is band-pass filtered between 0.5 Hz to 45 Hz. Different EEG amplitude transformation techniques are exploited to evaluate their influence on AD and healthy subject (HS) discrimination based on MFE. For each case, the EEG signals are divided in 3 s-epochs, and MFE is extracted as features and used in a binary classification task to distinguish between (HSs) and patients with AD. Different machine learning (ML) approaches are applied.

Figure 1 presents the key steps of the proposed approach: (1) preliminary data processing, (2) amplitude transformation, (3) MFE calculation and features extraction, and (4) classification. In more detail, EEG signals from patients with AD and HS recorded in the closed-eye resting state are used, as this condition is commonly adopted to minimize ocular artifacts and ensure cleaner baseline activity. The EEG signals are denoised for artifact removal and band-pass filtered. As the main focus of the present study is the amplitude transformation, different EEG amplitude transformation techniques are exploited to evaluate their influence on AD and HS discrimination based on MFE. In more detail, four amplitude transformation techniques are considered, with the case of no normalization serving as the reference. It is important to point out that this latter case refers to EEG data in which no explicit amplitude transformation is applied during preprocessing, thereby preserving the inherent variability introduced by the different acquisition choices. The effects of min-max normalization and standardization are investigated in two distinct configurations: global and single. In the global configuration, all channels are considered simultaneously, ensuring a uniform scaling factor. In the single configuration, each channel is considered separately. For normalizations, it is crucial to empirically choose an output amplitude range that ensures the symmetry of the EEG signal, typically with a zero mean, and obtain a unit standard deviation, analogous to standardization. For each case, the EEG signals are divided in 3 s epochs, and MFE is extracted as features and used to address a binary classification task to distinguish between healthy subjects (HSs) and patients with AD. Different ML approaches are applied including Support Vector Machines (SVMs), Random Forest (RF), and K-Nearest Neighbor (KNN).

In the following, the key steps of the proposed method are detailed.

### 3.1. Preliminary Data Processing

For each subject, the EEG signal is processed following two steps:

1. *Artifact Removal*: Independent Component Analysis (ICA) is applied to reduce artifacts in EEG signals [36], utilizing the FastICA algorithm. For component classification, IC labels are used, with an empirical rejection threshold set at 90%.
2. *Band Filtering*: A band-pass filter from 0.5 Hz to 45 Hz is applied to consider the most significant part of EEG signals [8]. More specifically, a finite impulse response (FIR) filter, with an order equal to the number of samples in a 3 s window, is used. Consequently, the first 3 s related to the transient are excluded from each EEG signal.

### 3.2. Amplitude Transformation

Initially, EEG data without amplitude transformation are examined to establish a reference for comparison. Then, the amplitude transformation techniques are applied to each preliminarily processed EEG signal. In particular, the following amplitude transformations approaches are alternatively employed:

- *min-max normalization* scales EEG data to an output range from $A_{min}$ to $A_{max}$. In this study, −5 to +5 is empirically chosen as $A_{min}$ to $A_{max}$, respectively, to ensure signal symmetry and a standard deviation of the order of 1, thereby maintaining the relative importance of each data point. This technique proves beneficial in EEG analysis by preserving the proportional significance of amplitude variations. For each EEG signals, the min-max normalization is applied on sliding windows of 3 s as follows:
  (1) $$x_{norm,i}=\left(5-\left(-5\right)\right)+\left(-5\right)\;\cdot \;\frac{x_{i}-x_{min}}{x_{max}-x_{min}}+\left(-5\right)=10\;\cdot \;\frac{x_{i}-x_{min}}{x_{max}-x_{min}}-5$$
  where $x_{norm,i}$ is the normalized signal, and *i* is the i-th channel. The $x_{max}$ and $x_{min}$ represent the median values of the maximum and minimum values across all windows for each subject. Thus, for each subject, the maximum and minimum values are determined for each window. Then, the median of all maximum values and the median of all these minimum values are calculated to obtain $x_{max}$ and $x_{min}$.
  This approach is implemented in two distinct manners:
  - *Single-channel normalization*;
  - *Global-channel normalization*.
  In the first case, $x_{max}$ and $x_{min}$ are computed separately for each channel, adjusting each channel to the same amplitude range. Conversely, in the case of global normalization, $x_{max}$ and $x_{min}$ are computed considering all channels simultaneously, preserving the amplitude ratio between channels. This is crucial for maintaining the integrity of cross-channel interactions.
- *standardization* transforms EEG data by centering the values around the mean and scaling them based on the standard deviation. This process ensures that the data have a mean of zero and a standard deviation of one, obtaining an output range based on the distribution of the data.
  Considering each EEG signal, the standardized signal is calculated as follows:
  (2) $$x_{stand,i}=\frac{x_{i}-x_{mean}}{\sigma }$$
  where *i* is the i-th channel, and $x_{mean}$ and σ represent the EEG mean and standard deviation for each subject, respectively.
  Also in this case, two distinct methods are considered:
  - *Single-channel standardization*;
  - *Global-channel standardization*.
  In single standardization, $x_{mean}$ and σ are separate for each channel. On the other hand, in global standardization, $x_{mean}$ and σ are calculated on all channels, maintaining the amplitude ratio between channels.

In the remainder of this manuscript, for the sake of brevity, the various amplitude transformation approaches are referenced as follows. *Reference* for the case without amplitude transformation: *Single Norm* and *Global Norm* stand for single-channel and global-channel min-max normalization, respectively; *Single Stand* and *Global Stand* stand for single-channel and global-channel standardization, respectively.

### 3.3. MFE Calculation and Feature Extraction

For each amplitude transformation approach, the EEG trace is divided into non-overlapping epochs of 3 s, ensuring an adequate number of samples for the subsequent MFE calculation. For each subject, the MFE is considered for each epoch and each channel. Specifically, MFE is computed by using an exponential fuzzy membership function. Regarding the MFE parameters, leveraging on literature [14,37], the values of m=2, n=2, and r=0.2×SD, where SD represents the standard deviation of the time series, were used. The whole formulation adopted for this study can be found in [17]. A range of scale factors from 1 to 20 is considered for MFE calculation. The scale factors refer to the multiple time scales at which the signal is analyzed. These scale factors control the extent of coarse-graining applied to the signal, which smooths it at each level to reveal its complexity at different resolutions. Previous studies indicate that 20 scale factors are necessary to effectively distinguish between the AD and control groups [14,37,38]. Additionally, the preliminary experimental results indicate that utilizing at least 20 scale factors is necessary to adequately capture the complete dynamics [17,24]. Consequently, at the end of this procedure, for each epoch, a total of 20 entropy values are yielded for each channel.

Then, the mean MFE for the long-scale factors (from 17 to 20) is calculated for each channel, resulting in one feature per channel. The decision to utilize only the long-term scales is based on preliminary analyses that indicated a more marked distinction between HS and AD at higher scales [17,24]. Furthermore, this strategy is beneficial in reducing the number of features for the subsequent classification task, thereby mitigating the risk of overfitting due to an excessively high number of features relative to the number of observations [39].

Finally, at the end of this phase, for each epoch of each subject, the number of MFE features corresponds to the number of channels.

### 3.4. Classification

To assess the impact of amplitude transformation on MFE features in discriminating patients with AD and HS, a binary classification task is addressed. For each subject, the *M* epochs are considered to be *M* independent observations. For each observation, *c* MFE features are obtained, where *c* is the number of considered channels. Each individual subject observation is labeled as AD or HS according to the subject’s condition.

The entire dataset is split into training and test sets, assuring the same number of HS and patients with AD in both sets. Different ML classifier are employed, namely SVM, RF, and KNN [40]. For each classifier, to identify the optimal architecture, a Leave-One-Subject-Out (LOSO) cross-validation approach with a grid search over different hyperparameters combinations is conducted (see Table 1).

Considering *N* subjects in the training set, in each LOSO cross-validation iteration, the model is trained on the observations from (N−1) subjects and validated on the observations from the remaining subject. For each iteration, to avoid potential data leakage, the training and the validation set were standardized on the mean and standard deviation of the training set (composed by the observations of (N−1) subjects). Once the best hyperparameters combination was identified, the classifier was retrained on all *N* subjects of the training set and tested considering the *K* subjects in the test set, standardizing the training and test set on the mean and standard deviation of the training set (in this case all *N* subjects).

The classification performance was assessed by calculating classification accuracy (Equation (3)) and MCC (Equation (4)). For each subject, the classification accuracy was evaluated individually; the classifier was provided with the *M* observations, resulting in *M* predictions per subject. The classification accuracy was calculated using the formula

(3) $$Class.\;Accuracy\;\left(i\right)\;=\frac{TP_{i}+TN_{i}}{TP_{i}+TN_{i}+FP_{i}+FN_{i}}$$

where *i* indicates the i-th subject, thus $TP_{i}$ = True Positive, $TN_{i}$ = True Negative, $FP_{i}$ = False Positive, and $FN_{i}$ = False Negative for the i-th subjects. In this context, TP values denote observations correctly predicted as AD, whereas TN values represent observations correctly predicted as HS. Then, a mean classification accuracy across all subjects in the test set was calculated along with its expanded uncertainty $k\sigma /\sqrt{N}$, where N is the number of subjects in the test set, σ is the standard deviation, and *k* is the coverage factor equal to 2 to establish a 95% confidence interval [41].

On the other hand, MCC is a statistical metric generally used for binary classification, and it takes into account all values of the confusion matrix. The MCC was calculated as follows:

(4) $$MCC=\frac{TP\;\cdot \;TN-FP\;\cdot \;FN}{\sqrt{\left(TP+FP)\;\cdot \;(TP+FN)\;\cdot \;(TN+FP)\;\cdot \;(TN+FN\right)}}$$

Basically, it returns good results only if all four values of the confusion matrix (TP, TN, FP, FN) are good. MCC ranges between −1 and 1. If MCC=1, all results are predicted correctly; if MCC=0, the model is unable to differentiate between the two classes, being close to random; if MCC=−1, all results are inverted, which is the worst-case scenario [42].

The MCC was computed globally using all the observations from all test subjects simultaneously. Therefore, the MCC was calculated considering all *M* observations from all *K* test subjects, resulting in a total of M·K observations. This approach provides an overall measure of classification performance across the entire test set.

## 4. Experimental  Approach

The following section details the experimental approach used for validating the proposed method. First, the EEG signals dataset used for the analysis is described. Then, the feature extraction and the classification task are explained.

### 4.1. Dataset

This section describes the datasets from which the EEG signals for AD and HS subjects were selected.

- *CAUEEG dataset* [43]: Collection of EEG signals recorded from 2012 to 2020 including subjects with different AD stages and healthy controls. Each EEG trace contains annotations regarding the subject’s age, event descriptions (closed and opened eyes, visual stimulation), and the diagnosis decided by the medical personnel. The data were acquired at a sampling rate of 200 Hz following the International 10–20 system for sensors placement.
- *TUH dataset* [44]: Collection of 26,846 clinical EEG signals acquired with different sampling rate (250 Hz, 256 Hz, 400 Hz, and 512 Hz) and following the International 10–20 system for sensors position. Each EEG record included a diagnostic report for the patient.

For the sake of clarity, it should be mentioned that the description contained in both the datasets states that the EEG recordings were acquired within hospital settings; hence, it is safe to reasonably assume that the instrumentation employed adheres to the established standards for clinical use.

From these two datasets, a total of 104 subjects were considered for the present study, comprising 52 AD patients and 52 HS individuals. The HS group consisted of 35 subjects from the CAUEGG dataset and 17 subjects from the TUH dataset, while the AD group comprised 35 subjects from the CAUEGG dataset and 17 subjects from the TUH dataset. Table 2 provides details on the subjects and their age ranges. In combining two public datasets, the variability introduced by the instrumentation is combined with the intrinsic variability between subjects. Considering the minimum continuous closed-eye segments of EEG signals available in the two datasets, for each subject, 51 s of resting-state EEG trace were selected by considering 19 EEG channels (sensor 10–20 system locations: Fp1, F3, C3, P3, O1, Fp2, F4, C4, P4, O2, F7, T3, T5, F8, T4, T6, FZ, CZ, and PZ) with linked earlobe referencing.

Additionally, in order to make the data uniform, the EEG signals, which were originally sampled at different frequencies, were resampled to 200 Hz (the lowest frequency in the two datasets). The resampling was performed using a polyphase implementation of interpolation and decimation. The interpolation and decimation ratios (p, q) are calculated by approximating the ratio between the desired and original sampling frequencies as a fraction of integers. An anti-aliasing filter is applied using a Kaiser window, with the cutoff frequency (fc) and transition bandwidth (df) calculated as fractions of the Nyquist frequency, and set by default to 0.9 and 0.2, respectively. This process was essential to ensure uniformity and consistent interpretation of the MFE, as it allows for the comparison of similar patterns at the same scale factor across all subjects.

The data were initially processed following the steps detailed in Section 3. The signal processing was carried out using *Matlab2023b*, specifically employing the *EEGLab* toolbox [45]. Firstly, Independent Component Analysis (ICA) was employed with an empirical rejection threshold set at 90%. Then, a band-pass filter was applied to isolate EEG signals within the frequency range of 0.5 Hz to 45 Hz. The amplitude transformation process was carried out, and finally, the EEG traces were subsequently segmented into epochs of 3 s, resulting in 16 epochs per subject.

### 4.2. Features Extraction and Classification

For each amplitude transformation approach, a total of 16 observations were obtained for each of the 104 subjects, resulting in a total of 1664 observations. For each observation, the MFE was computed by using the *Entropy Hub toolbox* [46]. Then, the average MFE for the long-scale factors (from 17 to 20) was calculated for each channel. This choice is based on the statistical analysis conducted and detailed in the Section 4.3. Consequently, a number of features equal to the number of channels were obtained. This resulted in a feature matrix of size 1664×19.

The subjects were divided into training and testing sets, with 74 subjects (1184 observations) allocated for training and 30 subjects (480 observations) reserved for testing. The testing set specifically included a mix of HS and AD subjects: 9 healthy CAUEEG subjects, 9 CAUEEG subjects with AD, 6 healthy TUH subjects, and 6 TUH subjects with AD. The selection of subjects for the test set was random to ensure a representative sample. As described in Section 3, the binary classification was addressed by employing SVM, RF, and KNN. For each classifier, the optimal hyperparameter configuration was determined by using a LOSO cross-validation method combined with a grid search. In each LOSO cross-validation iteration, the model was trained on the observations from 73 subjects and validated on the 16 observations from the remaining subjects. When the best hyperparameters combination was identified, the best model was retrained on all the training set (74 subjects) and tested on the observations from the 30 subjects in the test set. For evaluating the classification performance, classification accuracy and MCC were employed (see Equations (3) and (4)).

### 4.3. Statistical Analyses

MFE values and classification accuracy values were subjected to multiple statistical tests to ensure the robustness of the findings. For the MFE values of all 104 subjects, the analysis proceeded in two phases:

- For each amplitude transformation approach, a two-tailed *t*-test with FDR correction and a significance level of α=0.05 was applied to assess differences in the MFE values between the AD and HS groups, within each scale factor and channel. This analysis investigates variations in the discriminative power of the MFE depending on amplitude transformations. Additionally, the effect size was evaluated using Cohen’s *d* to further assess the magnitude of differences between the groups.
- In comparing amplitude transformation conditions, a mixed ANOVA was performed to examine the effects of scale factors in relation to the subject groups. To mitigate issues related to higher dimensionality, MFE values were averaged across all channels, resulting in 20 scale-dependent values for each group.

Regarding classification performance, statistical analysis was conducted on the accuracy values derived from the observations of the 30 test subjects across the three classifiers. Specifically, one-tailed *t*-tests with a significance level of α=0.05 were employed to compare the accuracy values between the *Reference* condition and each amplitude transformation, in order to assess whether any improvement in the classification accuracy was statistically significant.

## 5. Results and Discussion

For each subject (52 AD and 52 HS), a number of 16 observations (epochs) were obtained. For each amplitude transformation approach, the MFE was extracted channel-wise for each observation.

To ensure that amplitude transformations did not affect the intrinsic spatial structure of the EEG signals, an evaluation of inter-channel correlations was conducted. In more detail, Pearson correlation coefficients between EEG channels were computed for each amplitude transformation condition. No substantial differences were observed across the preprocessing methods, confirming that amplitude normalization does not alter inter-channel spatial relationships.

**Figure 2:**
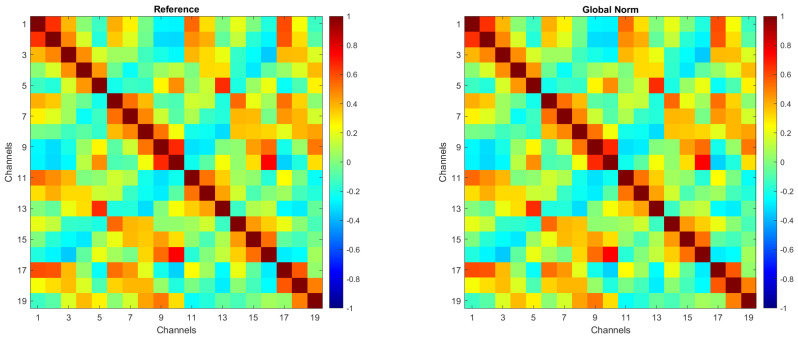
Correlation matrices for the *Reference* and *Global Norm* conditions, obtained by averaging the correlation matrices of 104 individual subjects.

For the sake of brevity, Figure 2 presents the correlation matrices for the *Reference* and *Global Norm* conditions. These matrices were obtained by averaging the correlation matrices from 104 individual subjects. Each matrix illustrates the correlation between EEG channels, with the color scale indicating the degree of correlation, ranging from −1 (negative correlation) to +1 (positive correlation). As shown, inter-channel correlations remain unchanged following the amplitude transformation.

### 5.1. Preliminary Results

**Figure 3:**
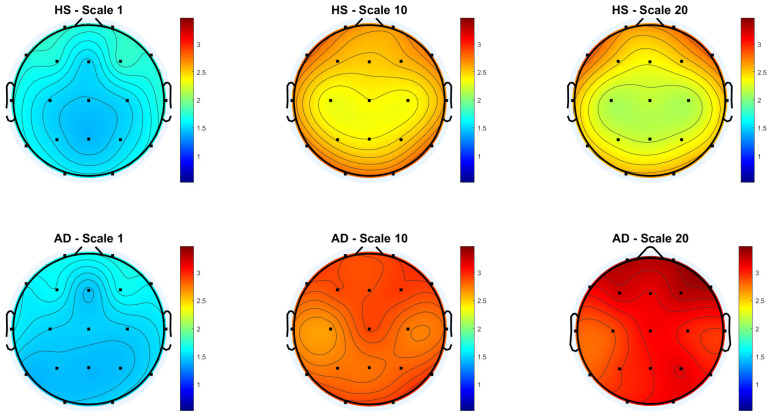
Topographic distribution of MFE values over the scalp for the Reference condition across the 19 EEG channels. The top row represents the HS group, while the bottom row represents the AD group. Each column corresponds to a different scale factor: first column—scale 1; second column—scale 10; third column—scale 20.

**Figure 4:**
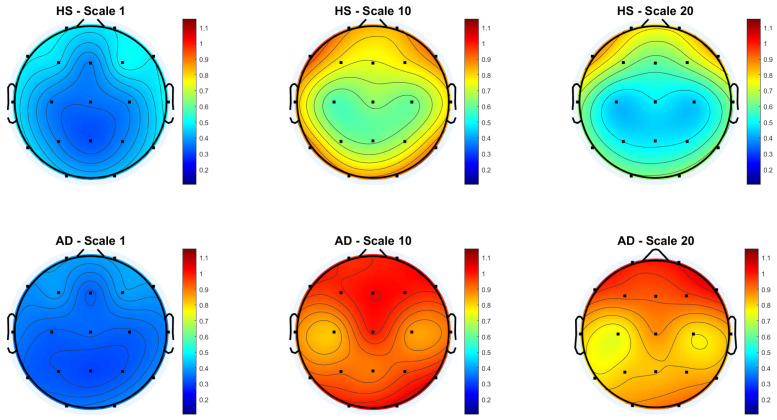
Topographic distribution of MFE values over the scalp for the Global Normalization condition across the 19 EEG channels. The top row represents the HS group, while the bottom row represents the AD group. Each column corresponds to a different scale factor: first column—scale 1; second column—scale 10; third column—scale 20.

Figure 3 and Figure 4 show the scalp topography of MFE mean values across 19 EEG channels under the *Reference* and *Global Norm* conditions, respectively, for HS (top row) and AD (bottom row) at three representative temporal scales (scale 1, 10, and 20), chosen for clarity. Under the *Reference* condition, both groups show similar low MFE values at scale 1. At scale 10, MFE values increase in both groups, more diffusely in AD. At scale 20, MFE values decrease in HS data, particularly in central and temporal regions, while they continue to rise in AD data, suggesting higher complexity. In the *Global Norm* condition, this trend is accentuated: AD shows a stronger and more widespread increase in MFE with scale, especially at scale 20, where differences between groups become more evident. Overall, MFE increases more consistently across scales in AD, highlighting group separation more clearly with normalization.

**Figure 5:**
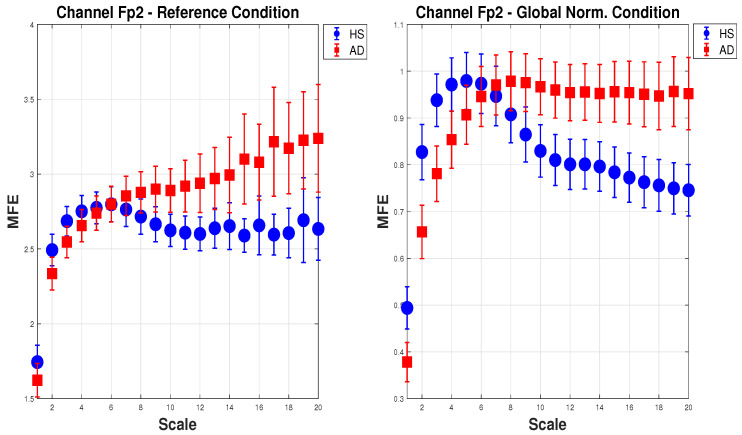
Mean and expanded uncertainty (k=2) of MFE for AD subjects (red squares) and HS (blue dots) for Fp2 channel across 20 scale factors. On the **left**, the MFE is calculated for the *Reference* condition, whereas on the **right**, for the *Global Norm*. As can be seen, *Global Norm* affects the MFE measures, enhancing the differentiation between HS and AD at all scales.

Figure 5 shows the mean of MFE for AD (red squares) and HS (blue dots) with the expanded uncertainty (k=2) calculated on 20 scale factors. For brevity, one channel (Fp2) in the configuration *(a) Reference* and *(e) Global Norm* is reported. In the *(a) Reference* configuration, as the scale increases, the MFE for HS increases slightly and then stabilizes, while the MFE for AD consistently increases, showing a clear separation between the two groups. In the *(e) Global Norm* configuration, the separation between HS and AD is more pronounced at lower scales, suggesting that *Global Norm* enhances the distinction between the two groups at all scales. This shows that *Global Norm* affects the MFE measures, particularly enhancing the differentiation between HS and AD. This may imply that *Global Norm* is more effective in capturing the entropy characteristics that distinguish the two groups.

**Figure 6:**
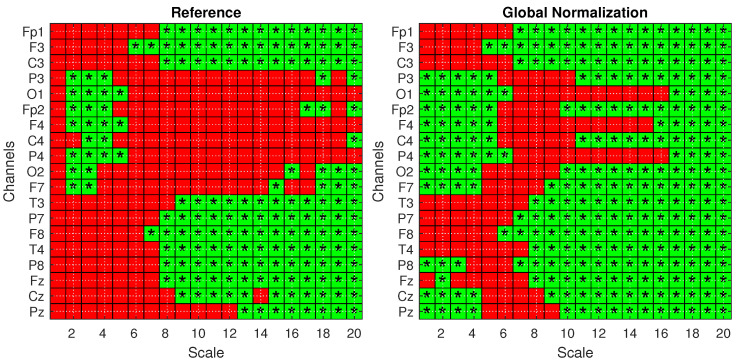
Statistical analysis with one-tailed *t*-tests with FDR correction for the *Reference* (**left**) and *Global Norm* (**right**) conditions. Green squares with an asterisk indicate statistically significant results (p<0.05) in distinguishing values from AD and HS subjects. The analysis is shown across 20 scale factors and 19 channels.

To confirm these findings and validate the significance of the observed differences, statistical analysis was performed according to Section 4.3. Figure 6 presents the statistical significance obtained from two-tailed *t*-tests, corrected for multiple comparisons using the FDR method, comparing the AD and HS groups under both the *Reference* (left) and *Global Norm* (right) conditions. Statistically significant results (p<0.05) are indicated by green squares with asterisks. A comparison of the two conditions reveals that the *Global Norm* leads to a broader detection of significant differences, particularly at higher scale values (scales 17 to 20) where all channels exhibit statistically significant differences. This suggests that global normalization enhances the sensitivity of the method in distinguishing between AD and HS subjects. In addition, the analysis reveals that, in all cases where the *t*-test results are statistically significant, the corresponding Cohen’s *d* values are consistently greater than 0.5, indicating medium to large effect sizes according to widely accepted conventions. A Cohen’s *d* value greater than 0.5 suggests that the difference between the groups is of medium magnitude or larger, supporting the practical significance of the observed effects rather than being attributed to random variation. Table 3 reports the *p*-values derived from a mixed ANOVA, evaluating the effects of scale factors, groups, datasets, and their interactions under different amplitude transformation conditions. The results confirm that the MFE values vary significantly across scales and between subject groups for all conditions. Notably, the effects are more pronounced under the *Global Norm* condition compared to the *Reference* condition, indicating a clearer differentiation between AD and HS groups by at least one order of magnitude.

Furthermore, the influence of the dataset factor, which reflects variability due to different acquisition settings, is significantly reduced following normalization procedures. This attenuation suggests that normalization strategies, particularly global normalization, enhance the robustness and generalizability of the results across heterogeneous datasets.

Overall, these findings further support the presence of distinct entropy patterns in AD and HS subjects, modulated by the scale of analysis and robust to variability in data acquisition protocols.

### 5.2. Classification Results

Following the proposed method detailed in Section 3, the average MFE for the long-scale factors (from 17 to 20) was calculated for each channels, obtaining 19 for each observation. As a results, a dataset of 104×16×19 was considered. The dataset was divided into two distinct sets to address a binary classification task: a training set and a testing set. After the hyperparameters search, the model was evaluated by considering the testing set comprising of 30 subjects. The results obtained on the test subjects are reported below.

#### 5.2.1. Classification Accuracy

**Figure 7:**
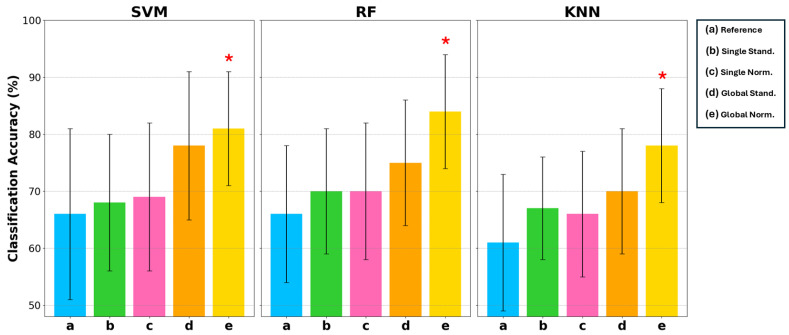
Comparison of classification accuracy in terms of mean and uncertainty (evaluated with a coverage factor equal to 2) on the test set among 3 types of classifiers: SVM, RF, and KNN. For each of these classifiers, 5 conditions are considered: *a* Reference, *b* Single Stand, *c* Single Norm, *d* Global Stand, and *e* Global Norm. The asterisk * indicates statistically significant differences with the Reference condition, considering α=0.05.

Figure 7 reports the mean classification accuracy on the test set associated with uncertainty, evaluated with a coverage factor of 2 to obtain a 95% confidence interval. For each classifier, the five different conditions of the input data are indicated by letters *a*, *b*, *c*, *d*, *e* representing the conditions of *Reference*, *Single Stand*, *Single Norm*, *Global Stand*, and *Global Norm*, respectively. Classification accuracy is reported on a scale of 50% to 100%, as 50% in binary classification represents complete randomness. Table 4 reports the results for each condition and classifier. These results indicate that *(e) Global Norm* consistently enhances the performance compared to other amplitude transformation techniques and the *(a) Reference*. As a matter of fact, *(e) Global Norm* achieved an average classification accuracy 81% and 84% with the SVM and RF classifier, respectively. The KNN classifier also achieved its best classification accuracy with the *(e) Global Norm* condition, reaching a mean accuracy of 78%. Although it did not achieve as high of an accuracy rate as SVM and RF, it still represents the optimal condition for KNN, showing a clear improvement over the other conditions. Moreover, it can be observed that classification accuracy increases on average and the uncertainty decreases when considering the *(a) Reference* compared to the amplitude transformations *b*, *c*, *d*, *e*, indicating less inter-subject variation when the signal is amplitude transformed.

Statistical analysis using a one-tailed *t*-test with a significance level of α=0.05 confirmed that the improvements in classification accuracy with *Global Norm* are statistically significant compared to the *Reference*. Statistically significant results are marked with a star symbol in Figure 7 and in bold in Table 4.

This suggests that *Global Norm*, maintaining the amplitude ratio between channels, is a more robust and reliable method for improving classification performance, thereby enhancing the overall reliability in distinguishing between AD patients and HS.

**Figure 8:**
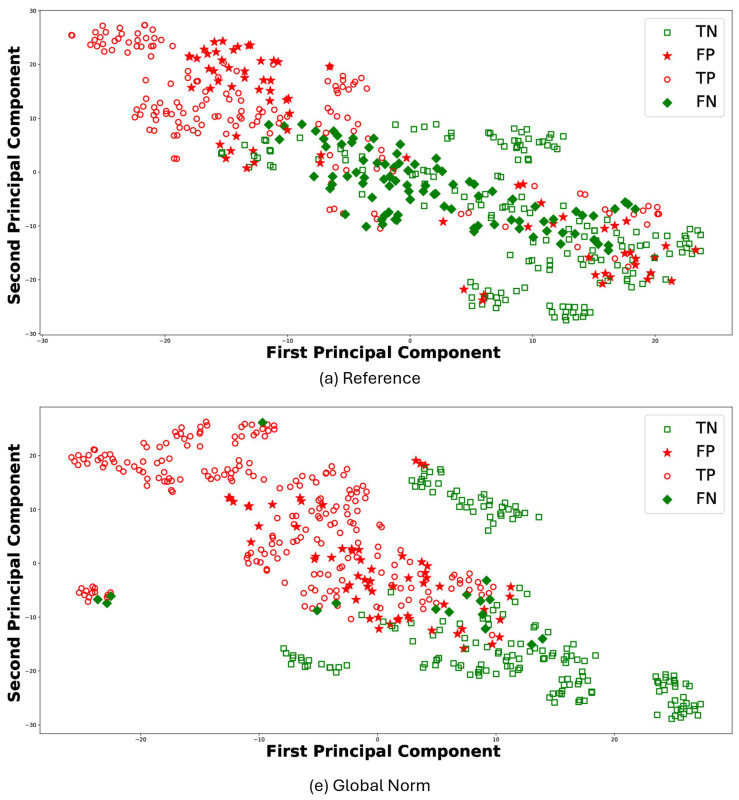
Scatter plots for RF classifier under the conditions *(a)* Reference and *(e)* Global Norm. In each case, TP are subjects correctly classified as AD, marked with a red dot, and TN are subjects correctly classified as healthy, marked with a green dot. FP are healthy subjects erroneously classified as AD, indicated with red stars, and FN are AD subjects erroneously classified as healthy, indicated with green stars.

For the sake of brevity, Figure 8 reports the t-Distributed Stochastic Neighbor Embedding (t-SNE) [47] for the results of RF classifier comparing *(a) Reference* and *(e) Global Norm*. t-SNE leverages the principal component analysis (PCA), identifying the principal components, in which the data variance is maximized. The t-SNE was performed using a perplexity parameter value of 30 and a single batch that included all features. Each plot visualizes the distribution of data points in terms of the first and second principal components: TP (empty circle), TN (empty square), FP (stars), and FN (diamond). In this context, TP values denote observations correctly predicted as AD, whereas TN values represent observations correctly predicted as HS. The t-SNE visualizations further support the quantitative findings. Under the *(e) Global Norm* condition, the data points representing true positives (TP) and true negatives (TN) are more distinctly separated compared to the *(a) Reference*, where there is more overlap. Specifically, most of the TP are located on the left side in *(e) Global Norm*, while the majority of the TN are on the right. In contrast, in the *(a) Reference* condition, these two classes do not separate as clearly, resulting in a more overlap between the data points of the two classes. Furthermore, the number of FP and FN decreases in the *Global Norm* condition compared to the *Reference*, further confirming that the *Global Norm* results in higher classification accuracy. The *Global Norm* condition better captures the underlying structure of the data, making the classes more distinguishable. This is particularly important for medical diagnoses, where accurate classification can significantly impact treatment decisions. Additionally, the reduction in FP and FN implies that the classifier is more reliable and robust when using the *Global Norm* condition. Lower FP means fewer HS are incorrectly classified as having AD, reducing unnecessary stress and medical expenses for those individuals. Similarly, lower FN means fewer AD subjects are misclassified as healthy, ensuring that more patients receive timely and appropriate treatment.

#### 5.2.2. Matthew Correlation Coefficient (MCC)

Table 4 also shows the MCC values obtained for the three classifiers (SVM, RF, and KNN) for the different conditions. The MCC for SVM shows a substantial increase when moving from *Reference* to *Global Norm*, indicating a significant improvement in the classification quality. The *Global Norm* condition has the highest MCC of 0.62. RF exhibits a similar trend to SVM, with a noticeable improvement in MCC from *Reference* to *Global Norm*. The *Global Norm* condition achieves the highest MCC of 0.70, suggesting that this condition enhances the classifier’s performance. KNN shows the lowest *Reference* MCC of 0.23. However, with the *Global Norm* condition, MCC increases to 0.58, indicating a significant enhancement. This demonstrates that the *Global Norm* method markedly improve the performance of KNN. Overall, across all classifiers, the MCC values consistently improve when moving from *Reference* to *Global Norm*, indicating that the *Global Norm* condition significantly enhances the classifiers’ performance, providing the most robust results. The improvement in MCC highlights the better handling of the balance between true positives and negatives versus false positives and negatives, leading to more reliable classification outcomes.

### 5.3. Discussion

The findings of this study, summarized in Table 5, demonstrate that transforming EEG signal amplitudes, particularly using the *Global Norm* condition, significantly enhances the ability to distinguish between AD patients and HS. The consistent improvement in classification accuracy and MCC across multiple classifiers (SVM, RF, and KNN) under the *Global Norm* condition highlights its robustness and effectiveness.

The *Global Norm* condition maintains the amplitude ratio between channels, which appears to capture the entropy characteristics more effectively, leading to a clearer separation between AD and HS groups. This is supported by both the quantitative results and the t-SNE visualizations, which show better class distinguishability and reduced false positives and negatives. These improvements are statistically significant and suggest that *Global Norm* provides a more reliable preprocessing technique for EEG data in the context of AD detection.

It is worth noting the reduction in uncertainty and inter-subject variability with amplitude transformation, indicating that these techniques make the EEG features more consistent across different subjects. This aspect is especially important for clinical applications, as it enhances the reliability of a diagnostic tool based on complexity measures such as MFE.

The study also highlights the potential for using relatively simple machine learning models (SVM, RF, and KNN) with carefully selected preprocessing techniques to achieve significant improvements in performance. This approach ensures that the findings are not limited to a specific model but are broadly applicable.

Furthermore, the methodology was validated using two separate, publicly available EEG datasets, thus ensuring greater generalizability of the results obtained. The data, acquired in different conditions with different instruments, show consistent improvements in classification accuracy and MCC, underscoring the robustness of the proposed preprocessing techniques when MFE is considered as a feature for AD detection.

However, the study has some limitations. The primary limitation of the current study is the relatively low classification performance, achieving a maximum accuracy of 84%, whereas some studies in the literature report up to 90% for binary AD-HS classification [29,48] and up to 80% for three-class classification, which includes mild cognitive impairment (MCI), an early stage of AD [49,50]. It should be noted that this work used two public datasets to generalize the approach, where the inherent variability between subjects is combined with the variability due to instrumentation. On the other hand, in a previous work [33], a classification accuracy of 93.6% was achieved on subjects from only TUH dataset.

The main objective of this research was to pursue a rigorous metrological approach for simple, common ML models. The significant challenge posed by the heterogeneity of EEG signals must be addressed to design effective clinical tools. Indeed, the results in Figure 5, Figure 6 and Figure 7 demonstrate that employing a standardized preprocessing approach to unify EEG data with varying characteristics effectively mitigates this heterogeneity. Specifically, the reduction in the uncertainty for the *(e) Global Norm* condition, compared to the *(a) Reference*, indicates greater consistency in entropy values across different subjects, thereby addressing inter-subject variability. Concerning intra-subject variability, the improved robustness is evidenced by generally higher classification accuracy, which reflects greater consistency between the temporally different observations of the same subject, achieved through signal amplitude transformation.

This study represents a first step towards the development of a generalized preprocessing pipeline for EEG signals. However, future work is required to ensure that this pipeline can be effectively applied to clinical data. Indeed, future studies will investigate the use of a larger number of subjects and epochs for each subject to improve the classification performance and enhance the robustness of the results. Moreover, future studies should also investigate the impact of comorbidities, as individuals with other known medical conditions were excluded from the current study to minimize potential confounding factors. Investigating comorbidities could provide a more comprehensive understanding for clinical practice. In addition, longitudinal studies will be crucial in understanding how EEG complexity measures evolve over time in AD patients, providing valuable insights into disease progression and the potential of EEG features to predict such changes. Finally, analyzing EEG complexity in response to specific stimuli, such as olfactory stimuli, may be of particular interest. Given that olfactory decline is one of the earliest symptoms of AD [51], this approach could improve the early diagnosis and understanding of AD and its early-stage MCI.

## 6. Conclusions

This study investigates different preprocessing strategies to make the calculation of MFE less dependent on the specific amplitude characteristics of the EEG signals at hand. Unlike previous works that applied preprocessing techniques for general EEG signal enhancement, this study systematically evaluates their role in making MFE a more robust and interpretable biomarker for Alzheimer’s disease. Indeed, the MFE formulation is inherently linked to signal amplitude, which can vary substantially among EEG systems, thus hindering the use of this metric for AD detection.

In light of this, the present study examined the impact of several amplitude transformation techniques on EEG signals with the objective of evaluating the effectiveness of MFE for the detection of AD using EEG signals.

In more detail, different amplitude transformation techniques, based on min-max normalization and standardization, were applied to EEG signals from patients with AD and HS. The impact of this transformation on AD detection was evaluated by extracting MFE as features and employing it in a binary classification task to distinguish between HS and AD using SVM, RF, and KNN. The robustness and general applicability of the proposed preprocessing techniques have been validated using two distinct, publicly available EEG datasets.

The results demonstrate that amplitude transformation is an effective technique for enhancing the distinction between AD patients and HS, as evidenced by the improved classification accuracy and MCC values. In particular, all classifiers demonstrate a mean classification accuracy exceeding 80% with a uncertainty of 10% when a global min-max normalization was applied. Furthermore, it can be observed that the classification accuracy increases on average and the uncertainty decreases when the *Reference* (case with no amplitude transformation) is considered in comparison to the amplitude transformation conditions. This indicates that there is less inter-subject variation when the signal is amplitude transformed.

In conclusion, this study highlights the importance of amplitude transformation of EEG signals in reducing intra- and inter-subject variability in MFE evaluation, generalizing and making more robust the adoption of this metric for AD detection. 

## Figures and Tables

**Table 1:** Hyperparameters and their values tested for each classifier. The table includes settings for SVM, RF, and KNN.

Classifier	Hyperparameters	Values
SVM	C	[0.1, 1, 10, 50, 70, 90, 100]
	gamma	[0.01, 0.05, 0.1, 0.5, 0.7, 0.9, 1]
	kernel	[rbf]
RF	n_estimators	[50, 100, 200]
	max_depth	[10, 20, None]
	min_samples_split	[2, 5, 10]
	min_samples_leaf	[1, 2, 4]
KNN	n_neighbors	[3, 5, 7, 9, 11]
	weights	[uniform, distance]
	metric	[euclidean, manhattan, minkowski]

**Table 2:** CAUEEG and TUH datasets, a summary of the characteristics of the chosen subjects.

Dataset	Class	Age (Mean ± Std)	N. of Subjects	Condition
CAUEEG	HS	77 ± 5	35	Resting state closed eyes
	AD	80 ± 5	35	Resting state closed eyes
TUH	HS	74 ± 6	17	Resting state closed eyes
	AD	81 ± 8	17	Resting state closed eyes

**Table 3:** Statistical significance from a mixed-design ANOVA assessing the effects of scale, group, dataset, and their interactions under different amplitude transformation conditions.

Condition	Scale	Group	Dataset	Scale × Group
Reference	<0.001	0.019	<0.001	0.003
Single Standardization	<0.001	0.446	0.216	<0.001
Global Standardization	<0.001	0.005	0.237	<0.001
Single Normalization	<0.001	0.002	0.011	<0.001
Global Normalization	<0.001	<0.001	0.363	<0.001

**Table 4:** Classification accuracy in terms of mean and uncertainty, and MCC values for three classifiers (SVM, RF, and KNN) under five different amplitude transformation conditions. The table presents the performance of each classifier, highlighting the effect of normalization methods on their ability to discriminate between AD patients and healthy subjects.

Classifier	Condition	Class. Accuracy (Mean ± Uncertainty) %	MCC
SVM	Reference	66±15	0.33
	Single Stand	68±12	0.37
	Single Norm	69±13	0.38
	Global Stand	78±13	0.60
	**Global Norm**	**81 ± 10**	**0.62**
RF	Reference	66±12	0.33
	Single Stand	70±11	0.41
	Single Norm	70±12	0.41
	Global Stand	75±11	0.50
	**Global Norm**	**84 ± 10**	**0.70**
KNN	Reference	61±12	0.28
	Single Stand	67±9	0.34
	Single Norm	66±11	0.33
	Global Stand	70±11	0.41
	**Global Norm**	**78 ± 10**	**0.58**

**Table 5:** Main findings and considerations.

Findings	Key Considerations
More separability and less uncertainty for MFE in *Global Norm* condition at all scales	Amplitude transformation allows to reduce inter- and intra-subject variability
(84 ± 10)% classification accuracy and MCC = 0.70 on 30 test subjects from two different public dataset	Amplitude transformation enables event simple ML models in discriminating AD and HS despite of high variability due to different subjects and instrumentation

## Data Availability

The original data presented in the study are openly available in caueeg-dataset at https://github.com/ipis-mjkim/caueeg-dataset (accessed on 5 June 2025) and in TUH EEG dataset at https://isip.piconepress.com/projects/tuh_eeg/ (accessed on 5 June 2025).
